# Comparative in vitro evaluation of contact activity of fluralaner, spinosad, phoxim, propoxur, permethrin and deltamethrin against the northern fowl mite, *Ornithonyssus sylviarum*

**DOI:** 10.1186/s13071-017-2289-z

**Published:** 2017-08-03

**Authors:** Bradley A. Mullens, Amy C. Murillo, Hartmut Zoller, Anja R. Heckeroth, Faris Jirjis, Annie Flochlay-Sigognault

**Affiliations:** 1 Department of Entomology, University of California, Riverside, CA 92521 USA; 2 MSD Animal Health Innovation GmbH, Schwabenheim, Germany; 3 Merck Animal Health, Madison, NJ USA

**Keywords:** Acaricide, Poultry, Control, Fluralaner, Spinosad, Phoxim, Propoxur, Permethrin, Deltamethrin, *Ornithonyssus sylviarum*

## Abstract

**Background:**

Northern fowl mites (*Ornithonyssus sylviarum*) are obligate hematophagous ectoparasites of both feral birds and poultry, particularly chicken layers and breeders. They complete their entire life-cycle on infested birds while feeding on blood. Infestations of *O. sylviarum* are difficult to control and resistance to some chemical classes of acaricides is a growing concern. The contact susceptibility of *O. sylviarum* to a new active ingredient, fluralaner, was evaluated, as well as other compounds representative of the main chemical classes commonly used to control poultry mite infestations in Europe and the USA.

**Methods:**

Six acaricides (fluralaner, spinosad, phoxim, propoxur, permethrin, deltamethrin) were dissolved and serially diluted in butanol:olive oil (1:1) to obtain test solutions used for impregnation of filter paper packets. A carrier-only control was included. Thirty adult northern fowl mites, freshly collected from untreated host chickens, were inserted into each packet for continuous compound exposure. Mite mortality was assessed after incubation of the test packets for 48 h at 75% relative humidity and a temperature of 22 °C.

**Results:**

Adult mite LC_50_ /LC_99_ values were 2.95/8.09 ppm for fluralaner, 1587/3123 ppm for spinosad, 420/750 ppm for phoxim and 86/181 ppm for propoxur. Permethrin and deltamethrin LC values could not be calculated due to lack of mortality observed even at 1000 ppm.

**Conclusions:**

Northern fowl mites were highly sensitive to fluralaner after contact exposure. They were moderately sensitive to phoxim and propoxur, and less sensitive to spinosad. Furthermore, the tested mite population appeared to be resistant to the pyrethroids, permethrin and deltamethrin, despite not being exposed to acaricides for at least 10 years.

**Electronic supplementary material:**

The online version of this article (doi:10.1186/s13071-017-2289-z) contains supplementary material, which is available to authorized users.

## Background

**Fig. 1:**
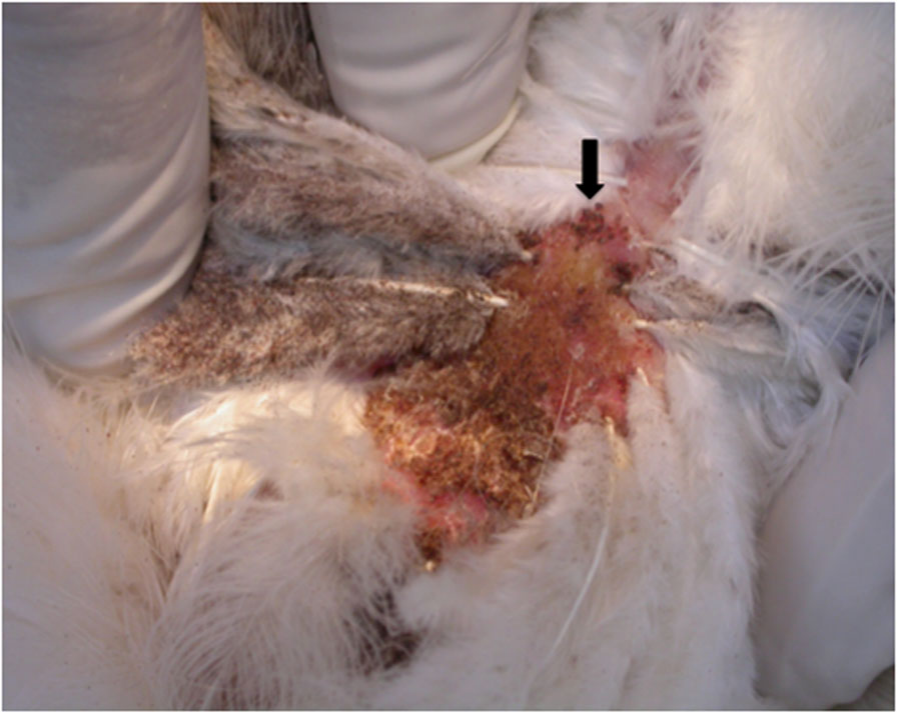
Northern fowl mites (*Ornithonyssus sylviarum*) on the vent region of an infested hen. A representative cluster of feeding mites indicated by an *arrow*

Two major ectoparasite species severely affect the poultry industry worldwide: the northern fowl mite, *Ornithonyssus sylviarum*, and the poultry red mite, *Dermanyssus gallinae* [1, 2]. Both mite species are obligate hematophagous parasites able to complete their life-cycles within about 1 week under optimal conditions [2–4]. Mite populations can become dense very quickly in commercial poultry facilities reducing hen performance and profitability [5, 6]. They differ mainly in that all stages of *O. sylviarum* mites live on the host full-time, occupying and laying eggs in the fluffy feathers mostly of the vent region [1] (Fig. 1), while *D. gallinae* lives predominantly off-host, hidden in cracks and crevices, and comes out nocturnally to feed on the birds [2]. Classical approaches to treat mite infestations mostly include the use of acaricidal sprays applied to the environment or to the host itself [6]. However, a complicating factor for both mite species is that they can persist without hosts for weeks and perhaps months in the environment [7, 8]. Their very small size makes them a difficult target for spray treatments and subsequent disinfestation of poultry houses between flocks. In addition, these acaricidal sprays must penetrate the feather layer from under the birds (vent region) to treat *O. sylviarum* on-host, which make it difficult to spray birds in enriched-cage or cage-free systems.

In North America, *O. sylviarum* is the most prevalent ectoparasite of commercial laying hen operations [1]. Control efficiency is threatened by serious mite resistance to a shrinking arsenal of acaricidal compound classes [9], especially for the synthetic pyrethroids, but also against carbamates and organophosphates. In the USA synthetic on-host control chemicals for *O. sylviarum* 15 years ago included the carbamate carbaryl, the organophosphate mixture tetrachlorvinphos/dichlorvos, and permethrin. Currently only permethrin is widely allowed for use, tetrachlorvinphos or its mixture with dichlorvos are used in some states, and carbaryl is no longer allowed [10]. Alternatives to traditional acaricides, such as botanical products or inert dusts, have been explored for both *O. sylviarum* and *D. gallinae* control, with inconsistent results; botanical products are notoriously variable, while silica dusts can be difficult to deploy effectively [2, 10, 11]. Entomopathogenic fungi, especially *Beauveria bassiana* and *Metarhizium anisopliae*, have promise for control of *D. gallinae* and perhaps of *O*. *sylviarum*, although results have been mixed [12–14]. It is critical that we explore other options, including new synthetic acaricides [2].

Twenty-first century developments of new classes of acaricidal compounds like isoxazolines, have restored optimism that safe and effective pest control could be maintained for crop, premise protection, and animal health. Isoxazolines work by binding to invertebrate GABA and glutamate channels [15], but act at previously unrecognized sites. This mitigates cross-resistance to other chemotypes, and differing target sites between arthropods and mammals result in selective toxicity and mechanistically based safety [16]. Isoxazolines, including fluralaner, afoxalaner and sarolaner, are under development and are of increasing importance in the control of external parasites in dogs and cats, including mites [17–20]. The present study was conducted to determine if fluralaner possesses contact activity against *O. sylviarum*, and compare this activity with that of other acaricides commonly used against mite infestations of poultry.

## Methods

The northern fowl mites used in the tests originated from a long-term colony maintained on a flock of untreated hens without any acaricide exposure for over 10 years at the University of California, Riverside Agricultural Operations property adjacent to the main campus (Animal Use Protocol A20150009, University of California, Riverside). The mites were originally collected from commercial hen infestations in California, between 2000 and 2005.

The larval packet test method, a common bioassay for ixodid tick larvae, was used for mite acaricide exposure [21, 22]. Whatman #1 filter paper (GE Healthcare UK Ltd., Thermo Fisher Scientific Inc., Waltham, Massachusetts, USA) was cut into 10 × 8 cm pieces. The filter paper pieces were labeled using a #2 graphite pencil according to the test material, dose, and replication number and were placed onto a larger piece of Labmat material (Bel-Art, Thermo Fisher Scientific, Inc., Waltham, Massachusetts, USA) which had a plastic backing to protect the lab counter from contamination. The filter paper rectangles were treated with compound solution of the acaricides (see below) and allowed to dry for 48 h before adding mites.

The carrier solvent, also used for the control packets, was a 1:1 mixture of 1-butanol (ACS reagent grade, 99.4% pure, Sigma-Aldrich Inc., Milwaukee, Wisconsin, USA) and pure olive oil (100%, Stater Brothers Markets, San Bernardino, California, USA). Fluralaner (10 mg/l) was supplied by Merck Animal Health, Summit, New Jersey, USA. Permethrin was purchased from Sigma-Aldrich Inc., Milwaukee, Wisconsin, USA. Other compounds were purchased from VHS Labs, Manchester, New Hampshire, USA. All compounds were used as technical grade acaricides.

The treatments tested included a carrier control and the acaricide treatments (i) fluralaner, 96.2% purity; (ii) spinosad, 94% purity; (iii) phoxim, 98% purity; (iv) propoxur, 99.5% purity; (v) permethrin, 98.8% purity; and (vi) deltamethrin, 99.5% purity. Stock solutions were prepared for fluralaner (1000 ppm), spinosad (4000 ppm), phoxim (2000 ppm), propoxur (1000 ppm), permethrin (1000 ppm), deltamethrin (1000 ppm). Preliminary tests (three replications per concentration) were done using the stock solutions and two sequential 10× dilutions (e.g. fluralaner at 1000 ppm, 100 ppm and 10 ppm) to establish approximate active ranges for northern fowl mites. Because the 1000 ppm solutions and below of permethrin and deltamethrin showed no mortality (see below), they were not tested further. Five concentrations over informative concentration ranges then were prepared by serial dilution from the stock solutions for the remaining test materials as follows: (i) fluralaner at 1, 2, 5, 10 and 20 ppm: (ii) spinosad at 200, 400, 1000, 2000 and 4000 ppm; (iii) phoxim at 250, 500, 1000, 1500 and 2000 ppm; and (iv) propoxur at 31, 63, 125, 250 and 500 ppm. Each filter paper received 800 μl of one of the test solutions by pipetting small drops of it evenly on the filter paper and allowing the liquid to absorb and evenly saturate the entire filter paper.

**Fig. 2:**
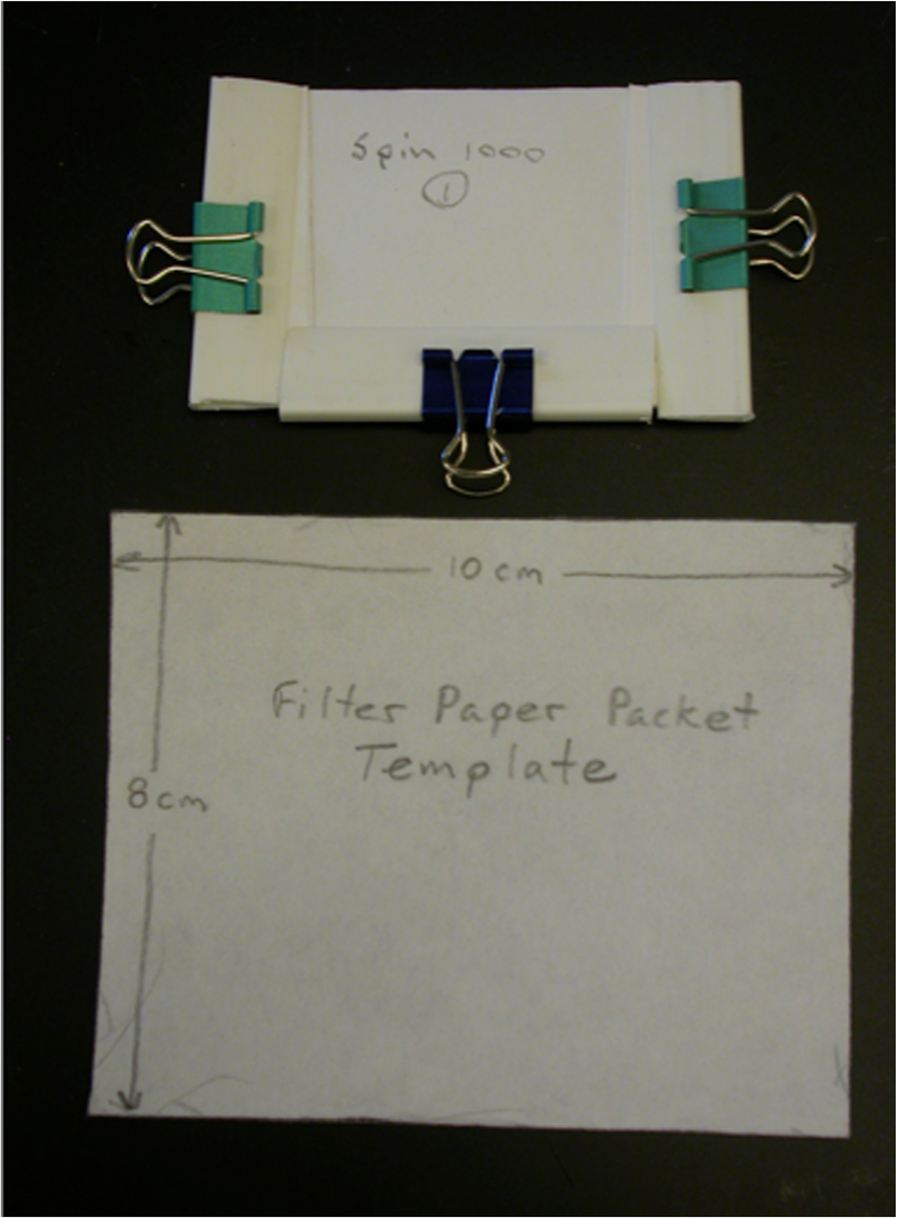
Test packets (clipped to enclose mites above and unfolded below) used to expose *Ornithonyssus sylviarum* to tested acaricides

Forty eight hours after treatment of the filter paper, mites were collected from hens by aspirating them from vent feathers into glass Pasteur pipettes [8]. Mites were transported back to the laboratory and exposed to the test packets within 3 h of removal from the hens. Mites were tapped from the pipettes onto a 15 × 15 cm metal sheet placed on an electronic chill table (Bioquip model 1431, Rancho Dominguez, California, USA) where they remained for a few minutes until the adult mites (largest life stage) were immobilized by cold and could be counted into the test packets. Packets (Fig. 2) were initially folded and then clipped closed on two sides, leaving one packet side open. Immobilized adult mites were added singly or in small groups through the open side of the test packets using a small paint brush. Test packets were placed on the chill table with the open end down during the mite counting process. Once in a packet the mites almost immediately warmed and became active, but moved quickly away from the chill table and toward the sealed (warmer) end of the test packet. This allowed more mites to be added to the bottom (colder side) of each packet until 30 mites per packet had been added. The last side was folded and clipped shut, enclosing the mites in the packet.

Mites were held in the packets on a screen support surface, placed in a plastic holding box, and positioned above a 2 cm deep layer of saturated NaCl solution. The lid was replaced in order to hold the relative humidity at a constant 75% inside the box [23], which is a comfortable humidity for the mites [8]. A separate plastic humidity box was used for each test material to avoid any cross-contamination. The mites were held for 48 h at approximately 22 °C (room temperature). After 48 h each packet was opened to assess the mite mortality. Adult mite mortality was scored under a dissecting microscope, and the adult mites (alive or dead) were removed using a paint brush. Mites were considered dead if they failed to move after gentle stimulation with a probe.

To minimize potential differences over time in mite condition as a factor, a single group of mites was usually assayed with several acaricides (plus a carrier control) on a given test day. However, time constraints limited testing to setting up or scoring 33 packets per day. There were always three carrier-treated controls, and those were used to correct for control mortality. Three compounds were tested at a time in addition to the control, with five concentrations and two test packets (replications) per concentration and compound. Each compound was evaluated in at least two full trials of this type. The fluralaner was evaluated in three full trials.

The mortality of mites tested for each compound concentration was calculated using the Henderson & Tilton formula [24]. If a treatment’s mortality was less than the control, it was counted as zero mortality.

$$ \% Mite\  Mortality=\left(1-\frac{n\  in\ Co\  before\  treatment\times n\  in\ T\  after\  treatment}{n\  in\ Co\  after\ tre atment\times n\  in\ T\  before\  treatment}\right)\times 100 $$

where *n* is the number of mites, T is a treated packet and Co is a control packet.

Statistical analyses were conducted using Minitab Version 17 (Minitab Inc., State College, PA, USA). The corrected % adult mite mortality was analyzed using probit analysis, which generated LC_50_ and LC_99_ values and 95% confidence intervals (CI) for each trial and acaricide. Lack of overlap in the 95% CI indicated statistically significant differences.

## Results

Preliminary testing for three concentrations over a 100-fold concentration range of the compounds tested showed the rough limits of activity. The results obtained in the range finding test led to the concentrations used for final testing. Control mite mortality was 20% in those tests. The two pyrethroid compounds showed negligible raw mortality even at 1000 ppm (the highest concentration tested), i.e. 12% for permethrin and 14% for deltamethrin. The pyrethroids were therefore not tested further. Of the remaining test compounds fluralaner showed 100% mortality at 1000 and 100 ppm, and 99% at 10 ppm. Mortality for spinosad was 98% at 4000 ppm, 29% at 400 ppm and 11% at 40 ppm. Phoxim showed 100% mortality at 2000 ppm, 20% at 200 ppm and 14% at 20 ppm. Propoxur had 100% mortality at 1000 ppm, 80% at 100 ppm and 10% at 10 ppm.

The adult mite mortality data caused by the five selected doses of each compound are shown in Table 1. Raw mite mortality data are supplied in Additional file 1: Table S1. There was generally good agreement between the trials for each tested compound. Control mortality for each trial varied from 6 to 19%. Most packets had the expected mite number. Since *O. sylviarum* are small and exact counts were done, a few packets experienced mite escapes. In those very few cases, the data from that packet were excluded from analysis, but remaining data were adequate to allow statistical analyses. In a few packets small numbers (< 5) of crushed mites (e.g. killed when the packet was folded closed) were excluded entirely from the analysis, reducing the total number of mites in that packet accordingly. In addition, if a treatment’s mortality was less than the control, it was counted as zero mortality.

**Table 1:** Corrected % adult *Ornithonyssus sylviarum* mortality at 48 h as a function of acaricide concentrations in test packets

Acaricide	Trial date	Dose (ppm)	Control mortality (%)	Corrected mortality (%)
Fluralaner	3-Apr-15	0	11.90	
		1		2.94
		2		34.98
		5		83.98
		10		100.00
		20		98.62
	20-Mar-15	0	5.60	
		1		40.52
		2		41.04
		5		92.86
		10		100.00
		20		100.00
	8-Mar-15	0	12.10	
		1		0.00^a^
		2		29.58
		5		81.04
		10		100.00
		20		100.00
	Trial Average	1		14.48
		2		35.20
		5		82.02
		10		100.00
		20		99.54
Spinosad	20-Feb-15	0	15.60	
		200		0.00^a^
		400		0.00^a^
		1000		25.00
		2000		78.29
		4000		95.92
	27-Feb-15	0	18.60	
		200		0.00^a^
		400		0.00^a^
		1000		20.83
		2000		83.88
		4000		100.00
	Trial Average	200		0.00
		400		0.00
		1000		22.92
		2000		81.09
		4000		97.96
Phoxim	20-Feb-15	0	15.60	
		250		0.68
		500		83.10
		1000		96.05
		1500		100.00
	27-Feb-15	0	18.60	
		250		6.88
		500		87.91
		1000		100.00
		1500		100.00
		2000		100.00
	Trial Average	250	3.78	
		500		85.51
		1000		98.03
		1500		100.00
		2000		100.00
Propoxur	20-Feb-15	0	15.56	
		31		0.00^a^
		63		44.70
		125		67.22
		250		100.00
		500		100.00
	27-Feb-15	0	18.60	
		31		1.67
		63		42.64
		125		89.76
		250		100.00
		500		100.00
	Trial Average	31		0.84
		63		43.67
		125		78.49
		250		100.00
		500		100.00

Trials were conducted in spring 2015 and used for probit analysis. Control packets indicated by “0 ppm” dose

^a^Treatments had less mortality than controls and are marked as no mortality

Table 2 shows LC_50_ and LC_99_ values. Fluralaner was the most active molecule, with an LC_50_ of 2.95 ppm and an LC_99_ of 8.09 ppm. Spinosad was the least toxic compound on an active concentration basis, with an LC_50_ of 1587 ppm and LC_99_ of 3123 ppm. Phoxim LC_50_ was 420 ppm and its LC_99_ was 750 ppm. Propoxur LC_50_ was 86 ppm and its LC_99_ was 181 ppm.

**Table 2:** Probit analysis results for tests of fluralaner and other tested acaricidal compounds tested against adult *Ornithonyssus sylviarum*

Acaricide	Trial	LC_50_ (ppm)	95% CI	LC_99_ (ppm)	95% CI
Fluralaner	1	3.48	3.06–3.93	9.87	8.81–11.32
	2	1.95	1.56–2.31	7.22	6.20–8.83
	3	3.41	3.12–3.73	7.18	6.50–8.14
	Mean	2.95		8.09	
Spinosad	1	1697	1562–1849	3595	3271–4026
	2	1477	1381–1579	2651	2418–2880
	Mean	1587		3123	
Phoxim	1	451	414–490	890	805–1015
	2	389	366–412	609	569–663
	Mean	420		750	
Propoxur	1	94	86–103	209	186–203
	2	78	72–84	153	140–171
	Mean	86		181	

## Discussion

The severe impact of *D. gallinae* infestations in the poultry industry, particularly in Europe, has led to several assessments of laboratory susceptibility and field efficacy of acaricides for its control, especially over the last two decades [2]. This is the first recent testing of acaricidal contact activity for *O. sylviarum*. Earlier bioassay-type laboratory testing has been conducted on single *O. sylviarum* isolates [25–27]. Extensive field surveys were done between 1999 and 2001 of acaricide susceptibility of 26 *O. sylviarum* field populations collected from commercial layer operations in southern California [9]. At that time *O. sylviarum* was already widely and extremely resistant to the class of synthetic pyrethroids (permethrin), and significant field resistance was noted (relative to a susceptible population) to representatives of the carbamates (carbaryl) and the organophosphates (the tetrachlorvinphos/ dichlorvos combination). Resistance issues are similarly serious for *D. gallinae*, and some level of resistance, or tolerance, exists for most classes of acaricidal active compounds, as reviewed by Abbas et al. [28].

Spinosad, a representative compound of the semi-synthetic class of spinosyns, is being marketed for the control of *O. sylviarum*, but in this study the compound had rather marginal contact activity for *O. sylviarum* relative to other compounds tested. Our calculated LC_99_ (3123 ppm) was approximately three times the commercial product label rate for *O. sylviarum* of 0.1% (1035 ppm) [29]. Several things should be noted, however. First, spinosad often has delayed toxicity against arthropods [30], although one recent study on *D. gallinae* showed high mortality by 2.5 h post-treatment [31]. Secondly, in vitro testing is not necessarily indicative of field efficacy, and we tested 48 h-old residues of spinosad on filter paper, as opposed to fresh applications against mites on a host. We are not aware of published in vitro or in vivo tests of spinosad against *O. sylviarum*. George et al. [31] showed 3–4 weeks activity of spinosad against *D. gallinae* at 1940–3880 ppm (treated metal plate residues) and suggested applying 3880 ppm (3.88 g/l) to the point of liquid runoff as a residual field application rate. Those authors also saw maximum effect at 2 weeks post-treatment and suggested spinosad activity was greater at high mite densities. The 3880 ppm rate [31] and the high use rate recommended for spinosad by the manufacturer for *Dermanyssus* (60 ml/7 l) [32] are above our estimated LC_99_ value for spinosad on *O. sylviarum*.

With a new mode of action and predominantly systemic activity, the isoxazoline ectoparasiticides have seen growing use in companion animals for the treatment of flea and tick infestations, and have been tested for a wide variety of blood-feeding arthropods [33], skin-dwelling mammal mites such as *Sarcoptes scabei* [17, 20] and a few non- blood-feeders such as blow fly or mosquito larvae [15]. The latter study showed the lower threshold for significantly increased mortality for most target arthropods occurred at somewhere around 1 ppm. It is difficult sometimes to discern whether toxicity was entirely based on ingestion (as it clearly was in some membrane blood-feeding tests) or whether fluralaner might also cause morbidity or mortality on contact alone.

A study by Williams et al. [34], showed likely in vitro contact activity of fluralaner against larvae of the brown dog tick *Rhipicephalus sanguineus*, but that was by immersion, which also could result in some small amount of oral/internal exposure. In the present study the mites should not have ingested any residues of fluralaner, except perhaps by grooming appendages that came into contact with it on the filter paper. The LC_99_ for adult *O. sylviarum* is only about 8 ppm.

## Conclusions

Northern fowl mites were highly susceptible to fluralaner by contact in this study. This mite resides primarily in the fluffy feathers of the vent region of chickens, where protonymphs and adults blood feed. This ectoparasite causes considerable economic damage to egg laying hens, caused by blood-feeding and the subsequent immune responses [6]. Pesticide sprays are currently the primary method of controlling *O. sylviarum*. The pesticides must be sprayed from underneath the birds at high pressures to effectively treat the mites living in the vent feathers. As birds are moved into alternative cages with solid floors and other structures, or cage-free environments, this type of treatment will become difficult to execute. Fluralaner has a higher contact activity than other acaricides we tested for *O. sylviarum*. This activity, together with its expected systemic activity, as demonstrated for it and other isoxazolines against other mite species [16, 19], would make fluralaner a valuable addition to the poultry mite control palette.

## Additional file

Additional file 1: Table S1. Raw northern fowl mite mortality data for trials of acaricides using filter paper packets. Second column has the pesticide and its concentration in ppm. *Abbreviations*: Cont, carrier control (butanol/olive oil); Spin, spinosad; Prop, propoxur; Phox, phoxim; Flur, fluralaner; Perm, permethrin; Delt, deltamethrin. (XLSX 52 kb)
